# RD Antigen Based Nanovaccine Imparts Long Term Protection by Inducing Memory Response against Experimental Murine Tuberculosis

**DOI:** 10.1371/journal.pone.0022889

**Published:** 2011-08-11

**Authors:** Mairaj Ahmed Ansari, Swaleha Zubair, Anjum Mahmood, Pushpa Gupta, Aijaz A. Khan, Umesh D. Gupta, Ashish Arora, Mohammad Owais

**Affiliations:** 1 Interdisciplinary Biotechnology Unit, Aligarh Muslim University, Aligarh, Uttar Pradesh, India; 2 Women's College, Aligarh Muslim University, Aligarh, Uttar Pradesh, India; 3 Molecular and Structural Biology Division, CDRI, Lucknow, Uttar Pradesh, India; 4 National JALMA Institute for Leprosy and OMD, Agra, Uttar Pradesh, India; 5 Department of Anatomy, JNMC, Aligarh, Uttar Pradesh, India; Institut Pasteur, France

## Abstract

**Background:**

The absence of certain genomic loci that are present in most of the virulent strains of *Mycobacterium tuberculosis* as well as lack of lasting memory responses are some of the major causes attributed to the non effectiveness of Bacille Calmette-Gue'rin (BCG) vaccine. Immunization schedules addressing these issues can offer better strategy for protection against tuberculosis.

**Methods:**

The immunological responses evoked upon administration of archaeosome based antigen delivery system comprising T cell antigen, Rv3619c (an ESAT-6 family protein), has been assessed against experimental murine tuberculosis in BALB/c mice.

**Results:**

Archaeosome based subunit vaccine has been found to elicit type-1 cytokines in the immunized mice. Besides effective T cell memory response, the Rv3619c based vaccine was able to reduce mycobacterial burden in the animals challenged with *Mycobacterium tuberculosis* infection.

**Conclusion:**

The data of the present study suggest that archaeosome encapsulated RD gene products offer substantial protection against *M. tuberculosis* infection.

## Introduction

Tuberculosis (TB) is a chronic infectious disease caused by *Mycobacterium tuberculosis* (*M. tb.*) and is one of the leading causes of mortality across the world [1], [2]. In fact, almost one-third of the humanity is inflicted with *M. tb*, wherein near about 25 million people are actively infected and 8.8 million new cases arising every year [2]. Upon infection with *M. tb* 10% individuals develop active disease within 1–2 years post exposure whereas remaining 90% individuals enter into latent infection state, which gets activated at a later stage of their lives when immunity deteriorates. The scenario becomes more complicated as ten million HIV patients are found to be co-infected with TB worldwide, which accounts for up to ∼33% mortality per year [3]. Furthermore, since the last global drug resistance survey, the prevalence of MDR TB in HIV infected patients, has risen up to 9% [4], [5].

Bacille Calmette-Gue'rin (BCG) is the only reliable, oldest and most commonly administered vaccine worldwide, which offers a balance between reduced virulence and preserved immunogenicity [6]. Although BCG appears to be effective at preventing disease in newborns and toddlers, however, its effectiveness in adults remains debatable. Besides other noticeable reasons, the variable efficacy offered by BCG vaccine is mainly attributed to the absence of certain regions in its genome and also because of its inability to boost long lasting memory in the host [7], [8].

Initially, eleven regions deleted from BCG (encompassing 91 open reading frames) were subsequently found to be present in *M. tb.* H37Rv strain. Recently, five additional regions with 38 ORFs have also been detected [9]. This is an evidence for the ongoing evolution of *M. bovis* BCG genome, since its original deviation leads to loss of important T cell antigens and considered to one of the possible reasons for non effectiveness of BCG against Mycobacterial infection in adults. Out of 16 deleted regions with different ORFs, we focussed on RD9, which is having 7 ORFs, i.e. Rv3617 to Rv3623 and recently demonstrated that Rv3619c, an ESAT-6-like protein (ESXV) with 94 amino acids, predominantly activates T cell response in the host [9]. In the present study, we made elaborated efforts to establish its potential as a candidate vaccine against experimental murine tuberculosis.

Traditional vaccines formulated with protein based Ags or dead microbes are internalized via endosomal compartment of antigen presenting cells and consequently evoke Ab production and, in general restricted for limited activation of Th cell responses. Like viruses, antibodies generally fail to work against most of the intracellular pathogens, including *Mycobacterium tuberculosis*. Infact, the only way to eliminate pathogen harbouring infected cells is their elimination by pathogen specific CD8^+^ cytotoxic and CD4^+^ T lymphocytes [10], [11]. With the identification of protective immunodominant proteins (*cf*. Rv3619c) that can be exploited as ideal targets in development of vaccines, there is an urgent need for efficient strategies capable of inducing long-term CTL responses.

It is imperative to identify and use right adjuvant or delivery platform for development of an effective subunit vaccine. Although co-administering Ags with certain immunostimulating adjuvants may facilitate a CTL response [12], [13], many of them possess undesirable side effects such as evoking severe inflammatory responses in the host, precluding their widespread use as human vaccines. Indeed, the only adjuvant currently approved universally for use in humans is aluminum hydroxide (alum), which is relatively weak inducer of cell-mediated immune responses [14].

There has been growing interest of using liposomes, the lipid based vesicles as carrier of antigens. We and others demonstrated that liposome prepared with lipid from lower microbes can elicit strong cell mediated and humoral immune responses against encapsulated antigen. Most of the organisms possess glycero-phospholipids with ester bond in their plasma membrane. On the other hand, the polar lipids in plasma membrane of various members of Archaebacteria share the constant-length phytanyl chains, usually fully saturated, and bonded by ether linkages to sn-2,3 carbon of glycerol backbone [15]. The distinct chemical structures of archaeal lipids confer considerable stability to the formed vesicular structures (archaeosomes) developed by using total polar lipids (TPL) of various archaebacteria including *Halobacterium salinarum* [15] The polar head groups exposed to the outer surface of archaeosomes have the potential to interact with mammalian cell surface molecules, whereas the type and proportion of lipid cores markedly influence the stability [16] and permeability [17] of the vesicular structures. Archaeosomes have been proven to be superior adjuvants, capable of facilitating strong and long lasting, CD4^+^ and CD8^+^ cytotoxic T cell and antibody responses against entrapped proteins in the host [18]–[20]. Because of the limitations of novel adjuvant based antigen delivery systems to evoke a CTL response, we evaluate herein the potential of archaeosome to facilitate presentation of exogenous Ags alongwith MHC class I molecules to induce desirable immune responses in the host.

Earlier efforts using various human T cell antigens such as ESAT-6, TB10.4, CFP-10, CFP-8 and CFP-15 (MTSP17) when administered in free form, or alongwith some adjuvants, failed to generate protective immunity against experimental murine tuberculosis [21]–[23]. The data of the present study suggest that archaeosome based vaccine imparts protective immunity against TB by activation of effector CD4^+^ and CD8^+^ T cells. Adjuvant property of archaeosome seems to enhance the vaccine potential of the Rv3619c, an RD family antigen, enabling it to offer strong protection against *M. tb* in Balb/c mice.

## Materials and Methods

### Reagents

All standard reagents used in the study were purchased from Sigma (USA), unless otherwise mentioned. The following reagents were procured from Difco Laboratories: Middlebrook 7H9 broth; Middlebrook 7H11 medium; and oleic acid, albumin, dextrose, and catalase (OADC). Tissue culture media (RPMI1640), Bovine serum albumin (BSA), antimycotic solution, and plastic-wares were purchased from BD Biosciences (USA).

pET expression vectors were procured from Novagen (Darmstadt, Germany). Oligonucleotides for gene isolation were bought from BIO Serve (Hyderabad, India). Restriction endonucleases, T4 DNA ligase and DNA size markers were procured from New England Bio-labs (Beverly, MA, USA). Taq polymerase, other reagents for PCR, Plasmid Miniprep kit, and the Gel extraction kit used for plasmid preparations and DNA purification processes, were obtained from Qiagen.

Nickel/nitrilotriacetic acid (Ni/NTA) superflow metal-affinity chromatography matrix was obtained from Qiagen. To concentrate expressed protein, Amicon-Ultra was used (molecular mass cut-off 3 kDa; Millipore, Bangalore, India). [^3^H]-thymidine was procured from Amersham Pharmacia Biotech.

### Antibodies

The following antibodies were procured from e-Biosciences: fluorochrome-labeled antimouse antibodies; fluorescein isothiocyanate–conjugated CD4 (GK 1.5), CD8 (53.67), phycoerythrin- conjugated CD44 (IM7), CD80 (B7-1), CD86 (GL1), PerCP conjugated CD62L (MEL-14) IgG2a (R35-95) isotype control. IgG1, IgG2a isotype kit (550487) and IL-4, interferon-γ (IFN- γ), IL-12 Cytokine kits were procured from BD Biosciences.

### *Mycobacterium bovis* BCG and *Mycobacterium tuberculosis* strain

*Mycobacterium bovis* BCG (Danish) and *M. tuberculosis* H37Rv strains were kindly provided by Dr. V.M. Katoch (National JALMA Institute for Leprosy and other Mycobacterial Diseases). *M. tb* was cultured into Middlebrook 7H9 broth containing 0.2% glycerol and 0.05% Tween-80 supplemented with albumin, dextrose and catalase [24]. The viability of the bacteria was determined by culturing them on Middlebrook 7H11 medium supplemented with oleic acid, albumin, dextrose, and catalase and counting the number of colony-forming units (CFUs).

### Cloning, expression and purification of Rv3619c

Genomic DNA of *M. tuberculosis* H37Rv was prepared and desired gene Rv3619c was cloned following method as described earlier [25]. Briefly, genes encoding Rv3619c were PCR amplified using oligonucleotide primers and *pfu* DNA polymerase, and cloned into pET-NH6 vector with restriction sites *EcoRI* and *HindIII*. This cloning strategy added an additional 30 residues at N-terminal including six residues of His-tag. The vectors containing Rv3619c were then transformed into BL21 (λDE3) *E. coli* cells and grown in Luria–Bertani medium supplemented with ampicillin (100 µg/mL).

For expression of protein, BL21 (λDE3) cells, containing the plasmid pETNH6-Rv3619c, were grown in Luria-Bertani medium supplemented with ampicillin (100 µg/mL) and induced at A_600_ = 1.0 with a final concentration of 0.5 mM isopropyl-β-d-thiogalactopyranoside. Culture was further grown for 12–14 h at 27°C. The protein of interest was purified over Ni/NTA matrix using a standard protocol under the denaturing conditions, as per the manufacturer's instructions except that NaCl and guanidine hydrochloride were excluded from the buffer. The eluted fractions were checked for purity by SDS/PAGE (15% gel) as well as Western blot analysis following the standard method [9]. The protein was refolded by dialysing it against refolding buffer containing 25 mM NaH_2_PO_4_, 100 mM NaCl and 1 mM 20 mM NaH_2_PO_4_, 50 mM NaCl, and 0.1% NaN_3_, pH 6.5. The construct pET-NH6-Rv3619c encoded protein contained 30 extra N-terminal residues with His-tag.

### Isolation of total polar lipids of Archaebacteria

The total polar lipids of *H. salinarum* were isolated following Bligh and Dyer method as modified in our lab [26]. In brief, the cells were cultured on nutrient agar plates containing 1 M NaCl. After 72 h, culture was harvested and washed thrice with phosphate buffer containing 150 mM NaCl. Subsequently, the cells were sonicated with the help of probe sonicator for 5 min (30 seconds pulse) and finally dispersed in mixture of 1∶2 V/V ratios of methanol and chloroform. The cell suspension was overnight stirred on magnetic stirrer at 4°C; and the solution was filtered to remove any solid residue. The filtrate was washed by gentle mixing with one fifth volume of 150 mM NaCl. The two phases (organic vs aqueous) were allowed to separate using separating funnel. Finally, the organic phase was evaporated using rota evaporator (Laborota-4000, Heidolph, Germany) to obtain the lipid.

### Preparation of liposome and assessment of antigen entrapment efficiency

To increase vaccine potential of T cell antigen, Rv3619c, we developed an archaeosome based antigen delivery system, where membrane lipids isolated from *H. Salinarum* were used for archaeosome preparation. Archaeal lipid based unilamellar vesicles were prepared by sonication method as standardized in our lab [27]. Briefly, dry lipid film was hydrated with normal saline to form lipid suspension. To get homogenous population of unilamellar liposome, hydrated lipid was sonicated for 1 h in bath type sonicator at 4°C (Power sonics, South Korea). Lipid vesicles were centrifuged for 10 min at 11,500 g at 4°C to remove large lipid aggregates [27]. The unilamellar liposomes were mixed with equal volume of protein solution (10 mg/ml stock). Several freeze thaw cycles were executed to increase the efficiency of entrapment. Un-entrapped protein was separated using sepharose 6B column. The Rv3619c bearing liposomes were eluted out in void volume. Entrapment efficiency was determined by Bicinchoninic Acid (BCA) method after lysing the liposomes with 1% Triton X-100. To rule out possibility that various observed immune responses evoked in the immunized animals are due to the archaeosomal total polar lipid contents only and not because of the antigen loaded liposomal structure of delivery system, we included plain archaelipid based vesicles that were not loaded with Rv3619c as a control throughout the study and termed them as “sham archaeosome.”

### Animals and immunization schedule

Female BALB/c mice (6–8 weeks old) were procured from JALMA institute for leprosy and other mycobacterial diseases, Agra, India. Mice were quarantined in the biosafety level 3 animal facility, in accordance with guidelines from the CPCSEA (Committee for the purpose of control and supervision of experiments on animals, Govt. of India), and kept on pellet feed diet and water *ad libitum*. Mice were immunized with various forms of Ag *viz*. free antigen, physical mixture of sham archaeosome and free antigen, archaeosomes entrapped antigen along with appropriate control groups of animals such as PBS (no adjuvant), sham liposomes *etc*. The animals were immunized by subcutaneous route at the base of their tail in lower abdominal region with 25 µg of Ag in a volume of 100 µl of vehicle per animal corresponding to lipid concentration in range of 1.5–2.0 mg/injection. On day 21, the animals were boosted with matching formulation of antigen using the same route of administration. In BCG group single dose of 1×10^6^ bacilli was administered intradermally.

#### Ethics Statement

All animal experiments were approved by the Institutional Animal Ethics Committees of IBU-AMU and NJIL & OMD, India. All animal experiments were performed according to the National Regulatory Guidelines issued by CPCSEA (Committee for the Purpose of Control and Supervision of Experiments on Animals). Our approval ID was 332/CPCSEA, Ministry of Environment and Forest, Govt. of India.

### Challenge with Mycobacterial infection and assessment of residual bacterial burden in vital organs

Twenty six weeks post last booster, 10 BALB/c mice from each group were challenged with virulent *M. tb* H37Rv through aerosol route. Suspension having a bacterial count of 5×10^7^/ml in a volume of 10 ml was added to the venture nebulizer unit of the middle brook Aerosol Generator device (Glas-col, USA) to deposit ∼100 bacilli in each mouse. To evaluate the protective efficacy of various in-house developed vaccine candidates, bacterial load in lungs and spleens of experimental animals was determined at various time points. After a stipulated time period (four and eight weeks post challenge), three animals from each group were sacrificed; their spleens and lungs were taken out aseptically and homogenized in 7H9 media. Different dilutions of prepared homogenate were plated onto 7H11 agar plates supplemented with oleic acid, albumin, dextrose and catalase. In BCG (Danish) immunized group, thiophene carboxylic hydrazide (TCH) at concentration of 2 mg/ml, was added to inhibit the growth of BCG and incubated for 3 weeks at 37°C. After stipulated incubation period, colonies were counted to calculate the bacterial load.

### Determination of antibody isotype in sera of immunized mice

Mice were bled at different time intervals and their sera were analyzed for the presence of antigen specific antibodies. Subsequently, the antibodies were analyzed for their isotypes using following method. Briefly, ninety-six well microtitre plates were incubated overnight with 200 ng antigen/well in carbonate-bicarbonate buffer (0.05 M, pH 9.6) at 4°C. After washing and blocking steps, the plates were incubated with serially diluted test and control sera at 37°C for 2 h. After excessive washing of the plates, 100 µl of (1∶5000 dilution of stock) goat anti-mouse anti- IgG1, and IgG2a antibodies were added in each well and incubated for 1 h at 37°C. After washing steps, 100 µl of (1∶5000 dilution of stock) HRP conjugated rabbit anti goat antibody was added to each well and each plate was incubated at 37°C for 1 h. The plates were washed again followed by adding 100 µl of substrate solution (6 mg OPD in 12 ml of substrate buffer with 5 µl of 30% H_2_O_2_) and were finally incubated at 37°C for 40 min. The reaction was stopped by the addition of 50 µl of 1 M H_2_SO_4_. The absorbance was read at 490 nm with a microtitre ELISA plate reader (Bio-Rad, USA).

### Isolation of T lymphocytes from spleens of immunized mice

Mice belonging to various immunized groups were sacrificed at different time points both post booster as well as post challenge with infection. Single cell suspension of the spleen was prepared as described elsewhere [28]. Briefly, spleens isolated from animals belonging to various groups were macerated and suspension was treated with ACK lysis buffer (0.15 mol/L ammonium chloride, 10 mmol/L potassium bicarbonate, and 88 mmol/L edetic acid) for lysis of the red blood cells. The cell suspension was centrifuged at 1500 g for 5 min and cell pellet was washed with HBSS solution 3 times and resuspended in RPMI 1640 medium containing 10% fetal bovine serum and 0.1% antimycotic cocktail.

### Lymphocyte proliferation assay

Lymphocyte proliferation assay was performed as described elsewhere [28]. Briefly, lymphocytes isolated from the spleens of mice belonging to various immunized groups were incubated in round bottomed 96-well plates (2×10^5^ cells per well) in 200 µL of RPMI 1640 medium with 10% fetal bovine serum. To determine the effect of concentration of antigen on T cell proliferation, varying concentrations (1–20 µg/well) of Rv3619c were used for priming of target cells. In the next set, splenic cells from various immunized groups were incubated with a known amount of corresponding matching formulations of Rv3619c. Cells incubated with the medium alone (without antigens) were used as controls. After 72 h, the cultures were pulsed with 0.5 µCi of [^3^H]-thymidine. After 16 h, the plates were harvested onto glass-fiber filter mats by the use of Tomtec-Harvester-96 (Tomtec). The incorporated radioactivity was measured with liquid scintillation spectroscopy (Wallac-1450 Microbeta Trilux; Perkin Elmer).

### Cytokine assay: Determination of IFN-γ, IL-4 and IL-12

Both type I and type II cytokines induced by splenocytes upon their co-culture in the presence of various forms of Rv3619c were estimated using appropriate and specific biotinylated antibody pairs according to the manufacturer's protocols. Briefly, 50 µl of the purified capture antibodies were adsorbed overnight on polystyrene microtitre plates at 4°C in carbonate buffer of pH 9.5. Plates were washed five times with PBST and blocked with 5% skimmed milk. After the usual steps of washing, 50 µl of the supernatant (isolated from cultured splenocytes after 48 h) was dispensed in each well to determine its cytokine content. After stipulated incubation time, the plates were thoroughly washed and incubated with biotinylated polyclonal goat anti-mouse cytokine detection antibody. Afterward, the plates were washed three times with PBST. Subsequently, 100 µl of streptavidin-HRP conjugate was added to each well and plate was incubated for 30 min. at room temperature. The plates were again washed three times with PBST and finally colored complex was developed with tetra methyl benzidine. The absorbance was read at 450 nm with a microtitre plate reader (Bio-Rad). A known specific recombinant cytokine was used as standard for calculating level of given cytokine in the samples tested and concentration was expressed as pg/mL.

### Expression of cell surface markers on immune cells as revealed by FACS analysis

The splenocytes were harvested and stained for flowcytometric analysis as described elsewhere [28]. Briefly, 1×10^6^ splenocytes were washed twice with FACS buffer (PBS with 1% BSA and 0.1% sodium azide). Cells were incubated with Fc block (2.4G2) or with FITC/PE/PerCP tagged monoclonal antibodies (CD4, CD8, CD44, CD62L, CD80, CD86 and isotype control) for 30 min at 4°C. After washing, cells were fixed with 1.0% paraformaldehyde. The cytometry data was acquired using a fluorescence activated cell sorter (GUAVA). Data was analyzed with Express-Plus software. The total number of cells of a definite phenotype (CD4^+^CD44^high^CD62L^low/high^, CD8^+^CD44^high^CD62L^low/high^) were calculated by taking the percentage of the gated cell population, as determined by flow cytometry, multiplying them with the total number of cells obtained per mouse, and finally dividing the furnished result by the number of events [29].

### Histopathology

Animals were sacrificed and their lungs were immersion fixed in 10% formalin, tissue blocks (of 3×5 mm dimensions) were processed for paraffin embedding, thereafter, 10 µm thick sections were cut with rotary microtome. Sections were subjected to conventional as well as Ziel-Neelsen staining to identify the acid fast bacilli. Stained sections were observed under light microscope (Olympus-BX 40-Japan). Observations were recorded in sample photomicrographs from Granuloma positive regions of the samples.

### Statistical analysis

Data were analyzed and two groups were compared employing the Student's t test as well as one way ANOVA (Holm-Sidak method) to compare all groups, using Sigma-Plot version 10.0 and 11.0 software. The *p* values<0.05 (*), <0.01(**), <0.001(***) were considered as significant for analysis of the data.

## Results

### Archaeosome entrapped Rv3619c antigen induces Th1 cytokines

**Figure 1:**
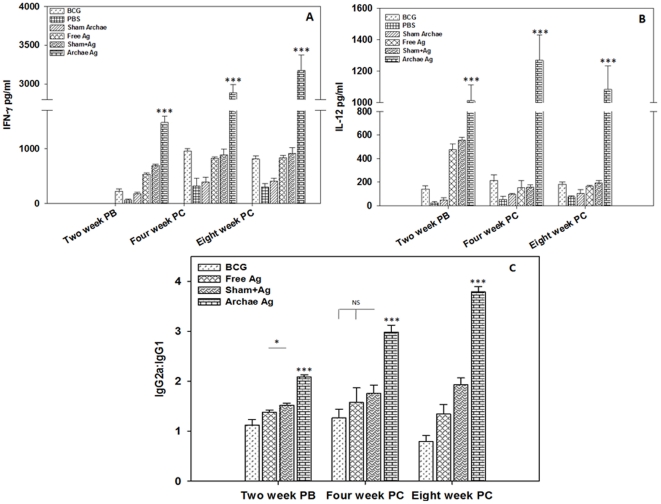
Archaeosome encapsulated Rv3619c induces type I cytokine expression in the immunized mice. Archaeosome mediated Th1/Th2 bias was ascertained by determining cytokine response in splenocyte culture supernatant belonging to various immunized groups at different time points; (**A**) IFN-γ, (**B**) IL-12. To activate splenocytes belonging to group of animals immunized with free Rv3619c, free form of 20 µg Rv3619c was used while lymphocytes isolated from group of animals immunized with physical mixture of sham archaeosome and Rv3619c were activated with physical mixture of 20 µg of free Rv3619c and sham archaeosome (∼1.5 mg archaebacterial lipid) splenocytes isolated from animals immunized with archaeosome entrapped Rv3619c group, were co-cultured with archaeosome entrapped Rv3619c (20 µg antigen). To further confirm the Th1/Th2 polarization upon immunization with archaeosome encapsulated Rv3619c, the sera of immunized animals were analysed for the presence of IgG2a and IgG1 isotype by sandwich ELISA method (**C**). The data represent mean of three determinants± S.D. and are representative of two different experiments with similar observation. Various immunized groups were compared to determine statistical significance of the data using student's t test analysis with *p*<0.05 (*), *p*<0.01(**), *P*<0.001(***) level of significance. PB (post booster), PC (post challenge), NS (not significant).

In the present study, we developed an archaeosome based antigen delivery system comprising Rv3619c, a T cell antigen. The recombinant protein was cloned, expressed and finally characterized by western blot analysis (**Figure S1**) before its use in development of nanoparticle based antigen delivery system. To establish Th1/Th2 bias of novel archaeosome based formulation, the presence of both Th1 (IFN- γ and IL-12) and Th2 (IL-4) type cytokines was assessed in splenocyte culture supernatant belonging to various immunized groups. The cytokine profile (**Figure 1**) clearly depicts that archaeosome entrapped Rv3619c induces significantly higher level of Th1 cytokines when compared to its free form, or its physical mixture with sham archaeosomes at various time points (*p*<0.001). Th2 (IL-4) response was insignificant at all time points post booster, but it was found to be increased by 4th and 8th week post challenge with Mycobacterium infection in all groups presumably due to onset of the disease (data not shown). There was comparatively less Th1 cytokine production in the animals, which were immunized with BCG when compared to archaeosome entrapped Rv3619c experimental groups post booster (**Figure S2**). While at 4 and 8 weeks post challenge time points, cytokine levels evoked by BCG were comparable with free as well as physical mixture of antigen with sham archaeosome. No significant expression of either Th1 or Th2 cytokines was detected in any of the control groups (PBS and sham archaeosome) at post booster when compared to immunized groups (*p*<0.001).

### Archaeosome mediated antigen delivery evokes predominantly IgG2a type antibodies in the immunized mice

To determine the antibody isotype switching, we evaluated Rv3619c antigen specific IgG1 and IgG2a response in serum of various immunized groups. As shown in **figure 1C**, archaeosome entrapped antigen had generated significantly increased levels of IgG2a than IgG1 at various time points (*p*<0.001). The IgG2a and IgG1 isotype ratio in BCG immunized group at post booster and 4th week post challenge time points remained near about 1, while at 8th week post challenge, antibody isotype response was somewhat biased towards IgG1. The isotype data suggest that archaeosome entrapped Rv3619c antigen induces better Th1 response in immunized animals when compared to other groups including BCG immunized group of animals.

### Archaeosome entrapped antigen stimulates T cell proliferation in a dose dependent manner

**Figure 2:**
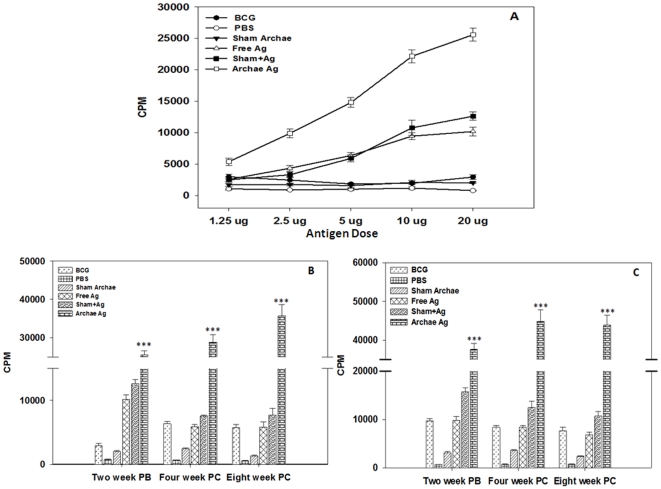
T cell proliferation response in various immunized groups upon stimulation with archaeosome encapsulated Rv3619c. (**A**) To determine the effect of amount of Rv3619c on proliferation of T lymphocytes; splenocytes, isolated from various groups of immunized Balb/c mice at two weeks post booster time point, were co-cultured in the presence of increasing amounts (1 to 20 µg) of Rv3619c Ag in flat-bottomed 96 well plates. After 72 h, [^3^H]-thymidine was added to each well and its incorporation in multiplying cells was measured after 16 h incubation with liquid scintillation spectroscopy. The accumulation of ^3^H, thymidine was determined in proliferating cells and denoted in term of counting per minute (cpm) values of stimulated cultures to represent Ag specific stimulation. (**B**) Proliferation of Rv3619c specific T lymphocytes isolated from immunized animals at various time points post booster and also at 4 and 8 weeks post challenge upon stimulation with a fixed amount (20 µg) of free Rv3619c. While (**C**) represents T cell proliferation in various immunized groups at various time intervals upon activation with corresponding matching formulations of Rv3619c. For stimulation of cells belonging to BCG immunized group, PPD at concentration of 20 µg was used, while maintaining rest of the incubation conditions same to that of archaeosome based cell groups. Similarly, for archaeosome encapsulated Rv3619c group, liposomised form of Rv3619c was used at a concentration of 20 µg (corresponding to ∼1.5 mg Archae lipid) to stimulate splenocytes. Splenocytes belonging to sham group were cultured in the presence of 20 µg of free Rv3619c, while splenocytes isolated from physical mixture group were incubated with empty sham archaeosome (∼1.5 mg lipid) mixed with 20 µg Rv3619c. Data represent the mean of three determinants ± S.D. Figures are representative of four independent experiments. The groups were compared with Student's t test to determine level of significance; *p*<0.05 (*), *p*<0.01(**), *p*<0.001(***).

The lymphocytes isolated from the animals immunized with archaeosome entrapped Rv3619c have higher T cell proliferation potential (37581±1589.15; CPM) post booster, when activated with matching archaeosome encapsulated form of antigen. There was statistically significant difference in T cell proliferation in archaeosome encapsulated antigen when compared with free as well as its physical mixture with sham archaeosome (*p*<0.001). On the other hand, when activated with free Rv3619c, T cell proliferation in BCG immunized group was 2926±340 CPM, while activation with PPD induced higher proliferation (9762.6±430 CPM) post booster in the same group (**Figure 2B and 2C**). Dose dependent proliferation was observed in both archaeosome entrapped (**Figure 2A**) and physical mixture of Rv3619c with sham archaeosome groups when cells were primed with free Ag as well as matching formulations of Rv3619c (data not shown). The lymphocytes isolated from the animals belonging to control group (immunized with PBS or sham archaeosome) did not induce considerable proliferation even at higher dose of the antigen. At the time points of 4^th^ (CPM counts for free antigen 28811.66±1934.59, and 44856.6±2935 for corresponding archaeosome encapsulated formulation) and 8^th^ (CPM counts for free antigen 35704±2958.40, and 43876±2584 for corresponding archaeosome encapsulated formulation) weeks post challenge, there was consistent increase in T cell proliferation when compared with other control groups (*p*<0.001). It is evident from the data shown in figure 2 that T cell proliferation was more prominent in the animals immunized with archaeosome encapsulated antigen even at 8^th^ week post challenge with infection.

### Flow cytometry analysis reveals increased expression of co-stimulatory and memory markers upon immunization with archaeosome entrapped antigen

**Figure 3:**
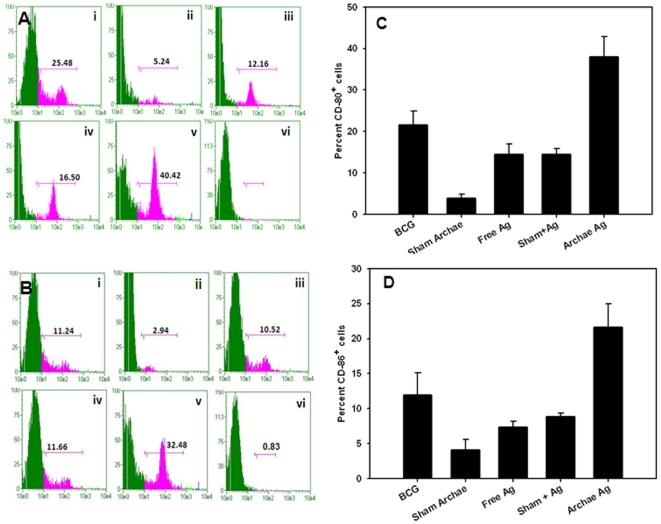
Archaeosome encapsulated Rv3619c upregulates costimulatory molecules on antigen presenting cells. Target cells were isolated following method as described in methodology section on 8^th^ week post challenge with *M. tb* infection. Co-stimulatory molecules, CD80 (B7-1) and CD86 (B7-2), were determined by staining of target cells with specific antibodies and subsequently analysed by flow cytometry. The histograms correspond to expression of CD80 (**A**), and CD86 molecules (**B**) on the surface of target cells. Various immunized groups are BCG (**i**) Sham (antigen free) archaeosome as control (**ii)** Free Rv3619c Ag (**iii**) Sham Archaeosome + free Rv3619c Ag; a physical mixture (**iv**) Archaeosome entrapped Rv3619c Ag (**v**) and isotype control (**vi**). The bar graphs corresponding to CD80 (**C**), and CD86 (**D**), depict the mean percent of three determinants (±S.D.). The expression data were analyzed with the Student's *t* test and are representative of 3 independent experiments. Archaeosome encapsulated Rv3619c Vs BCG *p*<0.01 (CD80), *p*<0.05 (CD86); BCG Vs physical mixture *p*<0.05 (CD80), *p* = NS (CD86); archaeosome Vs free Rv3619c Ag *p*<0.05 (CD80, and CD86).

The lymphyocytes isolated from various immunized groups of animals were stained with conjugated antibody markers specific for cell surface molecules and subsequently analyzed by flow cytometry. Expression of co-stimulatory markers CD80 (B7-1) and CD86 (B7-2), was higher in the group of animals immunized with archaeosome entrapped Ag when compared to its free form or BCG, on 8^th^ week post Mycobacterium challenge. Animals immunized with free as well as physical mixture of antigen expressed relatively less co-stimulatory molecules on APCs when compared to BCG. The histograms (**Figure 3**) clearly reveal the difference between archaeosome encapsulated Rv3619c versus sham archaeosome (without Ag) immunized animals.

**Figure 4:**
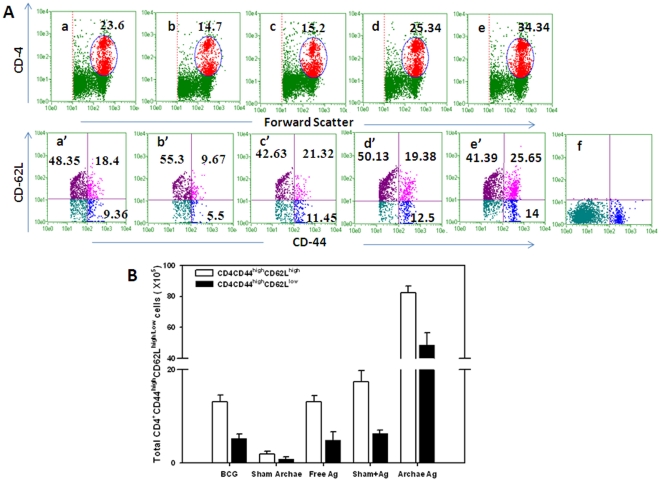
Augmentation of long-lasting CD4^+^ T cell (effector/central) memory response upon immunization of mice with archaeosome encapsulated Rv 3619c. After 8 weeks post challenge, the splenocytes were isolated from various immunized groups and analyzed for the presence of CD44^high^, CD62L^low/high^ on gated CD4^+^ T cells by FACS (**A**). The gated CD4^+^ T cells belonging to various immunized groups are denoted in the upper panel. Various experimental groups included in the study are: BCG (**a**) Sham (antigen free) archaeosome as control (**b**) Free Rv3619c Ag (**c**) Sham archaeosome + free Rv3619c Ag, a physical mixture (**d**) Archaeosome entrapped Rv3619c Ag (**e**) and isotype control (**f**). Lower panel indicates the analysed memory markers belonging to the corresponding CD4^+^ T cells in the upper panel. Bars indicate the total number of (CD4^+^ CD44^high^ CD62L^low^/^high^) (**B**). The data were analyzed with the Student's *t* test and are shown as the means (±S.D.) of 2 independent experiments. Archaeosome Vs BCG p<0.001 CD4^+^ (CD44^high^, CD62L^low^ and CD62L^high^); physical mixture Vs BCG *p* = NS CD4^+^(CD44^high^, CD62L^high^), *p*<0.01 (CD4^+^CD62L^low^); archaeosome Vs free Ag *p*<0.001 CD4^+^(CD44^high^, CD62L^high^, and CD62L^low^).

CD4^+^T cells isolated from mice vaccinated with archaeosome encapsulated Rv3619c Ag exhibited a typical effector memory (CD44^high^CD62L^low^) as well as central memory phenotype (CD44^high^CD62L^high^). Other control groups including BCG induced central memory to some extent on eight week post challenge. The population of effector memory phenotype in BCG immunized group was relatively less (**Figure 4A and 4B**). Interestingly, CD8^+^T cells isolated from archaeosome immunized group of animals showed effector memory (CD44^high^CD62L^low^) phenotype. On the other hand, the (CD44^high^CD62L^high^) central memory phenotype was also found to persist in animals immunized with archaeosome encapsulated Rv3619c. Importantly, effector memory phenotype was significantly higher in animals immunized with archaeosome entrapped Rv3619c, when compared to other groups such as BCG (*p*<0.01) and free form of Rv3619c (*p*<0.01), (**Figure 4** **and** **5**).

**Figure 5:**
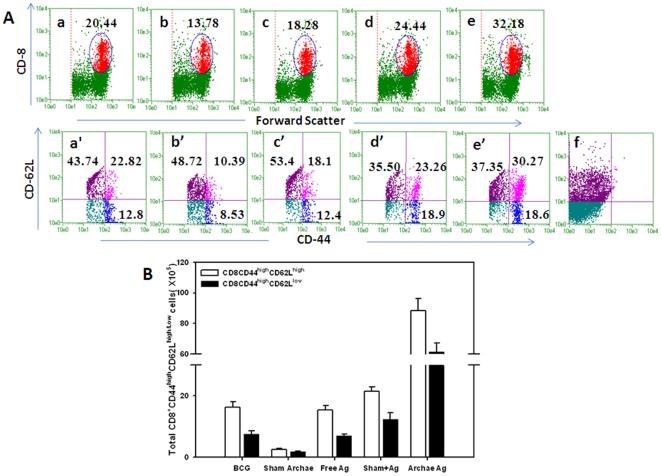
Expansion of the long-term CD8^+^ T cell effector and central memory response after immunization with various forms of Rv3619c on 8 weeks post challenge with *M. tb* Lymphocytes isolated from spleens of animals belonging to various immunized groups were stained for expression of CD44, CD62L, on CD8^+^ T cells and analyzed by flow cytometry. (**A**) The dot plot graphs in upper panel representing BCG (**a**), Sham (antigen free) archaeosome as control (**b**), Free Rv3619c Ag (**c**), Sham archaeosome + free Rv3619c, a physical mixture (**d**), Archaeosome entrapped Rv3619c Ag (**e**), and isotype control (**f**). The lower panel indicates the population of CD44 and CD62L^low/high^ on gated CD8^+^ T cells of corresponding upper panel. Bars indicate the total number of CD8^+^ T cells expressing CD44^high^CD62L^low/high^ (**B**), The data were analyzed with the Student's *t* test and are shown as the means (±S.D.) of 2 independent experiments. Archaeosome encapsulated Rv3619c Vs BCG p<0.001 CD8^+^ (CD44^high^, CD62L^low^ and CD62L^high^); physical mixture of sham archaeosome and free antigen Vs BCG *p*<0.05 (CD8^+^CD44^high^, CD8^+^CD62L^low^), *p* = NS (CD8^+^CD62L^high^); archaeosome encapsulated Rv3619c Vs free Rv3619c Ag *p*<0.001 CD8^+^ (CD44^high^, CD62L^low^ and CD62L^high^).

### Protective efficacy of archaeosome entrapped Ag against Mycobacterial infection

**Figure 6:**
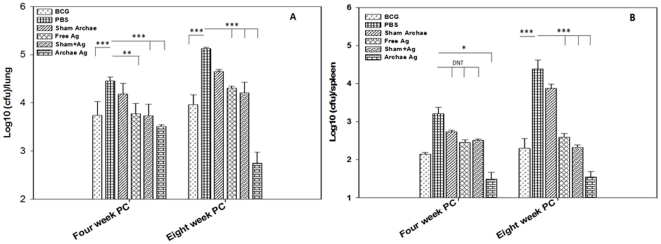
Archaeosome encapsulated Rv3619c offers protection against *M.tb* infection in BALB/c mice. Mycobacterial load, in the lungs (**A**) and spleens (**B**) of vaccinated mice, belonging to various groups of animals, was enumerated by plating tissue homogenates of lungs and spleens of vaccinated mice followed by counting the numbers of colony-forming units (CFUs) at 4 and 8 weeks post challenge following method as described in methodology section of the text. The data were expressed as means of three determinants ± S.D. and are representative of 4 independent experiments. Statistical analysis was performed by ANOVA (analysis of variance) using Holm-Sidak method; *p<*0.01(**), *p<*0.001(***).

Protective efficacy of various vaccine preparations was evaluated on the basis of their potential to suppress bacterial load in lungs and spleens of the immunized mice. Histopathology of lungs, at 4 and 8 weeks post challenge, was also used as a parameter to assess prophylactic potential of various vaccine preparations against experimental tuberculosis. As shown in **figure 6**, archaeosome entrapped Rv3619c antigen showed significant reduction in bacterial burden in immunized animals when compared to BCG, free Rv3619c, and its physical mixture with sham archaeosomes (*p*<0.001). The BCG vaccination stands protective when compared with free form or physical mixture of the antigen with sham archaeosome at 4 week post challenge. On 8th week post challenge, the mycobacterial load was log_10_ 2.75±0.23 lower in the group of animals immunized with archaeosome entrapped Rv3619c when compared with that of PBS control group. Interestingly, while archaeosome entrapped Rv3619c showed near about log_10_ 1 reduction, while other immunized groups showed increase in CFU count in lungs on 8^th^ week post challenge when compared to bacterial burden at 4^th^ week post challenge (*p*<0.001). In comparison to PBS control, archaeosome entrapped Rv3619c exhibited a significant reduction in CFU count (*p*<0.001), which corresponded to log_10_ 2.38, while BCG, free Rv3619c and its physical mixture with sham archaeosome vaccinated mice showed only log_10_1.16, log_10_0.82 and log_10_0.92 reduction respectively (*p*<0.001) (**Figure 6A**). The residual bacterial load data clearly establish superiority of archaeosome entrapped Ag in eliminating tuberculosis, than other control immunized groups.

Archaeosome entrapped Ag again showed significant reduction of mycobacterial load in the spleens of immunized mice as well when compared to control (PBS only) group on 4 week post challenge (*p*<0.001). Although 8th week post challenge BCG also showed reduced bacterial burden but not as significant as in archaeosome entrapped Ag. Interestingly, archaeosome encapsulated antigen clearly suppresses splenic bacterial load more effectively when compared to BCG immunized animals (*p*<0.001) (**Figure 6B**).

**Figure 7:**
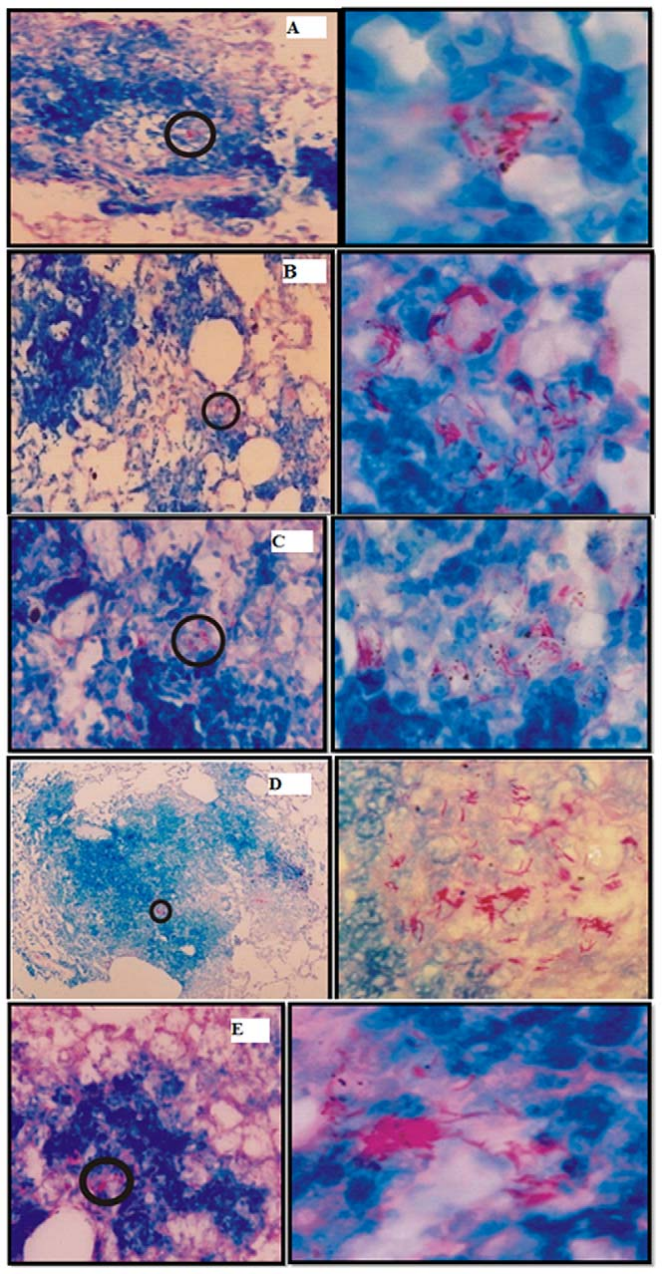
The histopathological study of lungs obtained from animals belonging to various immunized groups. At 8 weeks post challenge, the lungs obtained from animals belonging to various immunized groups were fixed in formalin, to prepare them for section cutting. Subsequently, sections were stained with hematoxylin and eosin or Ziehl-Nelson stain to facilitate acid fast staining of bacilli as described in materials and methods section. Various experimental groups included in the study are: BCG (**A**), Sham control (**B**), Free Rv3619c (**C**), physical mixture of sham archaeosome and Rv3619c (**D**) and archaeosome entrapped Rv3619c (**E**). The panels on left side represent low power photomicrographs (200×; except D which is 100× of original picture) of sections while right side panel represents the high magnification (1000×) of the inset part of corresponding left panel. Acid fast bacilli can clearly be seen in higher resolution photomicrographs.

We also examined the histopathological changes and the presence of acid-fast bacilli in the lungs of the infected animals, to track progression of the disease. While animals immunized with BCG, free Ag, its physical mixture with sham archaeosome and other control groups developed typical granulomatous lesions accompanied by massive perivascular and peribronchiolar cuffing, in contrast, animals immunized with archaeosome encapsulated antigen had relatively less granulomatous lesions. Furthermore, lesser consolidated lungs, having more normal alveolar structures with presence of relatively less acid-fast bacilli were observed in the archaeosome entrapped Ag immunized group on 8^th^ week post challenge with infection (**Figure 7**).

## Discussion

Keeping into consideration the fact that successful suppression of tuberculosis can be achieved by employing T cell mediated immunity in the host, various human T cell antigens have been identified and their immunostimulatory potentials have been established in model animals [30]. As poor efficacy of BCG, a widely used vaccine across the globe, is attributed to deletion of certain major genes from its genome during its passages to progeny, we anticipated that supplementing one of the deleted gene products of BCG could control tuberculosis. Consequently, in the current study, we used Rv3619c, a T cell antigen, that belongs to deleted RD-9 region of BCG as a vaccine candidate and amplified its prophylactic potency by its entrapment in archaeosome based antigen carrier.

The data of the present study suggest that immunization protocol employing archaeosome entrapped antigen,

- strongly upregulates Th1 type cytokines (IFN-γ and IL-12) in the host;
- produces higher ratio of IgG2a type antibodies to IgG1 in the sera of immunized animals (Figure 1 and Figure S2);
- induces antigen specific T lymphocytes (Figure 2);
- helps in upregulation of costimulatory molecules (CD80/CD86) on the surface of APCs (Figure 3);
- induces CD4^+^ and CD8^+^ T cells with effector (CD44^high^CD62L^low^) as well as central memory (CD44^high^CD62L^high^) phenotype (Figure 4 and 5);
- reduces mycobacterial burden in vital organs of infected mice (Figure 6).

The higher efficacy of archaeosome encapsulated Rv3619c preparation could be explained on the premise that besides depot formation, encapsulation of antigen in archaeosome helps in its slow and steady release in the surrounding milieu. The specific composition of the archaeal lipid not only helped in slow and sustained release of the antigen from the archaeosome for longer time duration but also facilitated specific uptake of antigen loaded archaeosomes by both macrophages and dendritic cells; potential antigen presenting cell types of the host. It seems that liposome harbouring macrophages as well as dendritic cells act as secondary depots that help in disseminated distribution of antigen to various lymphoid organs of the body. Earlier, Krishnan *et. al.* [20] reported that CTL response in immunized mice lasted for a substantially long period following immunization that involved administration of archaeosome encapsulated model antigen. Parallel to their report, we found remarkable potential of archaeosome encapsulated Rv3619c to activate host cells even after a period of six months. This exhibits the capability of Rv3619c encapsulated archaeosomal formulation to induce a stable immunological imprint, in the form of antigen specific CD4/CD8 T lymphocytes, that enables the host to mount an accelerated response later, favouring its development as a candidate vaccine.

As shown in figure 1, archaeosome entrapped Rv3619c antigen induces predominantly Th1 (IFN-γ, IL-12) response in the immunized animals. BCG, on the other hand, in a manner similar to free and physical mixture of antigen with sham archaeosomes induces moderate levels of IFN- γ and IL-12 post booster. Moreover, the BCG induced Th1 biased immunological responses get suppressed post challenge with infection. Besides, the concentration of type II cytokines viz. IL-4 was found to increase in all other control groups post challenge with *M. tb* presumably because of the spread of infection (data not shown). The cytokine profile data clearly indicate that archaeosomes entrapped Rv3619c antigen induces significantly high levels of Th1 cytokines consistently when compared to other control immunized groups.

The archaeosome entrapped Rv3619c was also successful in inducing higher IgG2a response when compared to IgG1 in the immunized animals post booster. The higher IgG2a level was maintained post challenge with *M. tb.* infection in the group of animals that was immunized with archaeosome encapsulated Rv3619c. In case of BCG vaccinated group, IgG2a∶IgG1 ratio was almost equal post booster but it decreased at 8th week post challenge. Interestingly, both free as well as physical mixture of antigen with archaeosome (sham) showed upregulated IgG2a ratio, although, it was not as significant as in the case of the animals immunized with archaeosome entrapped antigen (**Figure 1C**). It has been earlier reported that B cell education offered by IFN-γ producing Th1 cells results in IgG2a production whereas predominance of Th2 cells favours IgG1 response [34]. The isotype switching data indicate that archaeosome mediated upregulation of IFN-γ instructs B cell for production of IgG2a type of antibodies mainly. Both cytokine profile as well as antibody isotype data suggest that archaeosomes entrapped Rv3619c has bias towards Th1 response, a requisite for effective prophylaxis against tuberculosis.

Lympho-proliferation studies further support Th1 bias of the delivery system as archaeosome entrapped Rv3619c Ag induced T cell proliferation in dose dependent manner (**Figure 2A**). Among various forms, only archaeosome entrapped Ag had induced consistently increasing T cell proliferation, whereas free and physical mixture of Ag induced relatively lower T cell proliferation which decreased further upon challenge with infection. As earlier studies have ruled out involvement of Toll like receptors in archaeosome mediated activation of immune system, the observed feature could be attributed to efficient targeted delivery of Rv3619c to macrophages and dendritic cells supposedly mediated by mannose residues present on their surface [19]. Besides, we speculate that mannose specific targeting of archaeosomes to macrophages and dendritic cells would be helpful in downsizing the dose of antigen required to get desirable immune response and thereby making the vaccine cost effective. Surprisingly, data of the present study suggest that BCG, inspite of lacking of RD gene products including Ag Rv3619c was found to evoke Rv3619c specific immune response in the host (**Figure 2**). The observed T cell proliferation can be explained on the premise that some proteins in BCG have overlapping peptide sequences to that of Rv3619c and were successful in inducing production of cross reactive T lymphocytes in the immunized animals. Recently, Gareth *et. al*. have also reported that certain cross reactive epitopes of Rv3619c were found to be recognized by animals immunized with *M. bovis* [35].

Activation of naive T cells requires signaling through the Ag specific TCR and co-stimulatory molecules. The best-characterized co-stimulatory pathway is due to interaction of CD28 on T cells with CD80 (B7-1), CD86 (B7-2) on APCs [31]. We found maximum upregulation of both CD80 and CD86 in archaeosome vaccinated animals when compared to BCG, free Ag, or physical mixture (Figure 3). Although CD4^+^ T cells play crucial role in prophylactic action against *M. tb.*, however CD8^+^ T cells are also equally needed for the same, especially during the chronic phase of infection [11]. There was a substantially greater percentage of effector memory phenotype (CD44^high^CD62L^low^) as well as central memory phenotype (CD44^high^CD62L^high^) on both CD4^+^ and CD8^+^T cells belonging to animals immunized with archaeosome encapsulated antigen. In general, central memory persists after rapid clearance of acute infections, and is more effective in controlling secondary infections involving intracellular pathogens [32]. On the other hand, the effector memory was reported to be induced by chronic infections [33], [34]. This fact is clearly suggestive of the continued low-level presentation of Ag to both the CD4^+^ and CD8^+^ T cells by APCs at later time points and thus predicts an Ag-depot effect offered by archaeosomes, which in turn results in producing a balanced central and effetor memory in the host.

Lastly, we assessed the role of archaeosome entrapped Ag Rv3619c in suppressing the mycobacterial burden in two vital organs viz. lungs and spleen of the experimental animals. The data of present study show that only archaeosome entrapped Ag significantly reduces bacterial load in lungs of immunized animals at 4^th^ week post challenge and this was further reduced on 8^th^ week post challenge, whereas other immunized groups showed significantly high bacterial burden 4^th^ week post challenge which increased further on 8^th^ week post challenge (**Figure 6A**). Similarly, in the case of spleen, lower mycobacterial count was observed in archaeosome entrapped Rv3619c Ag, while other immunized groups showed elevated levels of mycobacterial burden in spleens of challenged mice (**Figure 6B**).

The data of the present study clearly suggest that archaeosome based Rv3619c subunit vaccine confers long term protection against tuberculosis in model animals. We speculate that the delivery system can be made more efficacious by development of antigen bearing liposomes loaded with more than one type of potential antigens (*cf.* RD antigens) that would suffice shortcomings of the BCG vaccine.

Finally, we infer that the subunit vaccines employing RD gene product could be of great importance as potential prophylactic measure against tuberculosis. The vaccine would be having wider scope in subjects who had been earlier immunized with BCG under routine immunization protocol followed by boosting with RD gene based subunit vaccine products that can evoke desirable immune response to eliminate *Mycobacterium tuberculosis* infection.

### Conclusion

In the present study, immunoprophylactic potential of archaeosome encapsulated Rv3619c (T cell antigen of ESAT-6 family protein) has been established against *Mycobacterium tuberculosis*. We have examined cellular as well as humoral responses to evaluate the efficacy of the subunit vaccine. The following major findings have emerged from this study: Archaeosome entrapped Rv3619c Ag successfully helped in augmented proliferation of T cells; predominant production of Th1 cytokines (IL-12, IFN-γ) and production of antibody predominantly of IgG2a isotype response; up-regulated expression of co-stimulatory (CD80 and CD86); and memory markers (CD44^high^, CD62L^low/high^) on CD4^+^ and CD8^+^ memory T cells; reduction in the mycobacterial burden; and the lung pathology. To the best of our knowledge this is the first report showing higher efficacy of archaeosome loaded Rv3619c based vaccine against experimental tuberculosis.

## Supporting Information

Figure S1 Western blot analysis of recombinant Rv3619c expressed in BL21 (λDE3) using pET-NH6 vector. The expressed recombinant Rv3619c was subjected to 15% SDS PAGE and transferred to nitrocellulose membrane. The membrane was blocked with 2% BSA in Tween PBS and finally incubated with polyclonal sera developed in mice. Lane 1: depicts protein marker, Lane 2: expressed recombinant protein Rv3619c.
(TIF)

Figure S2 Cytokine response in splenocytes culture supernatant from various immunized groups at post booster on different time points. Archaeosome mediated Th1/Th2 polarization was ascertained by determining various cytokines at different time point post booster; (**A**) IFN-γ, (**B**) IL-12. To determine the antibody response IgG2a to IgG1 ratio was depicted using sandwich ELISA method (**C**). The data represent mean of three determinants± S.D. and are representative of two different experiments with similar observation. Statistically two groups were compared using t test analysis with *p*<0.05, *p*<0.01, *p*<0.001 level of significance. PB stands for post booster. For IFN-γ and IL-12 archaeosome entrapped Rv3619c Vs BCG *p*<0.01(eight week post booster), *p*<0.05 (sixteen week post booster); IgG2a∶IgG1 ratio *p*<0.05 (four week post booster, eight week post booster) and *p* = not significant (12 week and 16 week post booster).
(TIF)
