# Supplementary figures and images for: RD Antigen Based Nanovaccine Imparts Long Term Protection by Inducing Memory Response against Experimental Murine Tuberculosis
Source: PLoS One. 2011 Aug 11;6(8):e22889. doi: 10.1371/journal.pone.0022889 (PMC3154911; doi:10.1371/journal.pone.0022889)

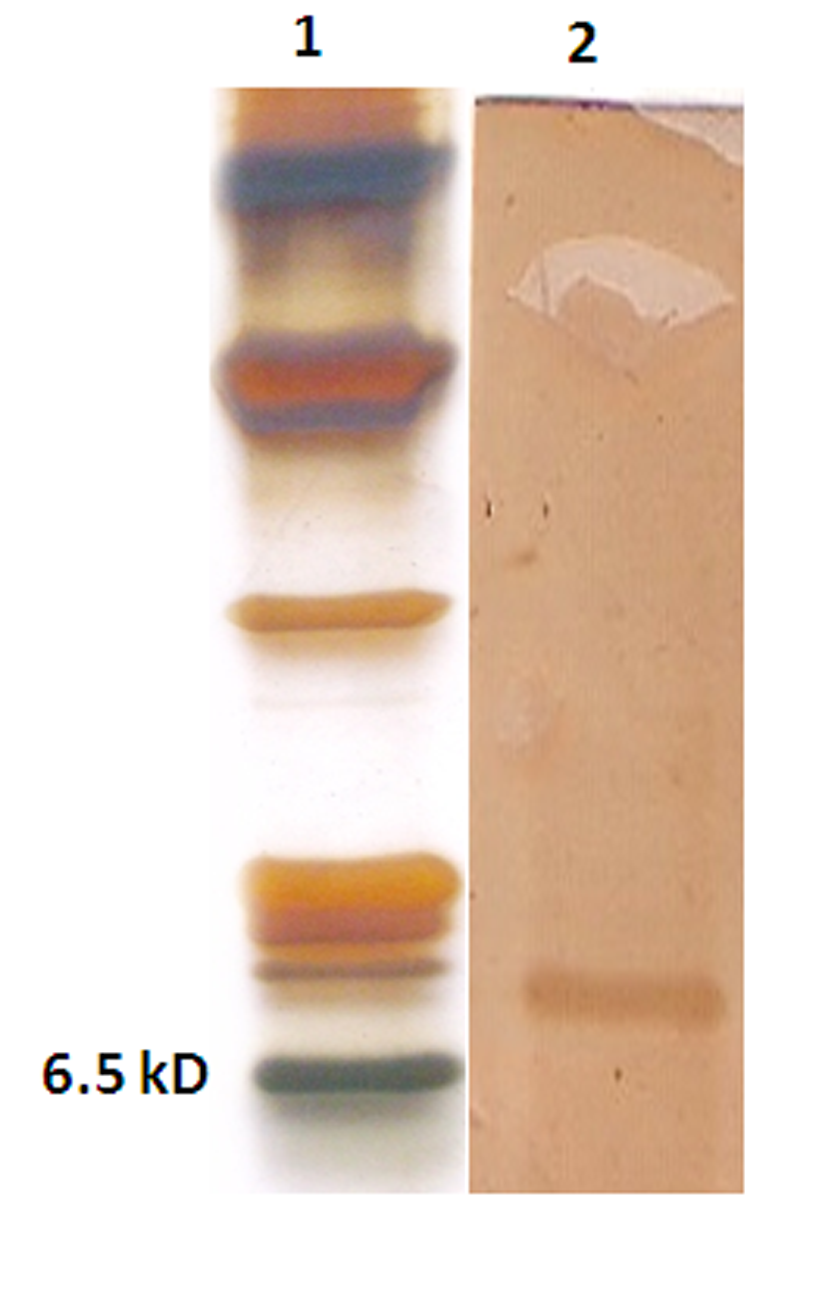

Supplement: Figure S1 — Western blot analysis of recombinant Rv3619c expressed in BL21 (λDE3) using pET-NH6 vector. The expressed recombinant Rv3619c was subjected to 15% SDS PAGE and transferred to nitrocellulose membrane. The membrane was blocked with 2% BSA in Tween PBS and finally incubated with polyclonal sera developed in mice. Lane 1: depicts protein marker, Lane 2: expressed recombinant protein Rv3619c. (TIF) [file pone.0022889.s001.tif]

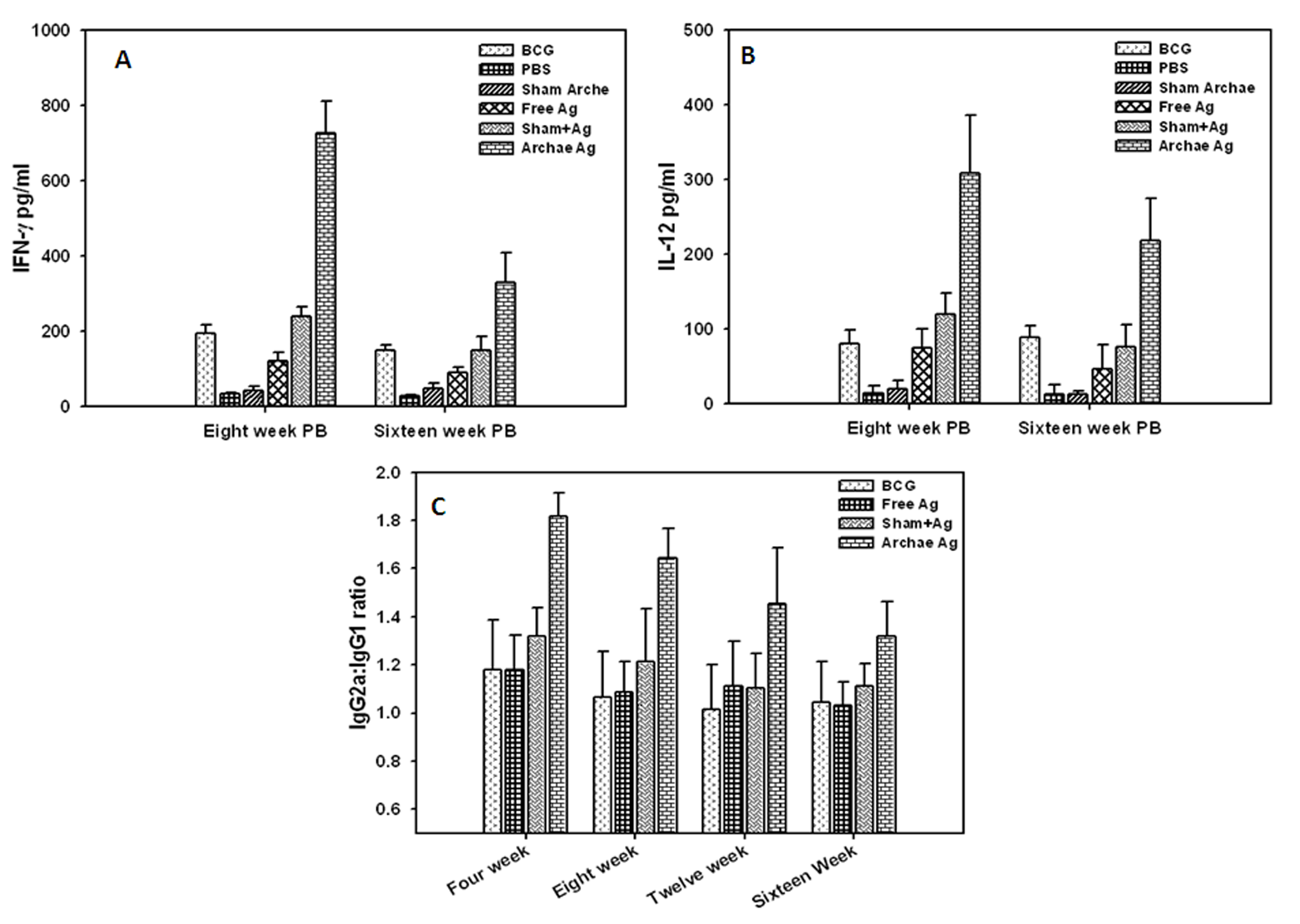

Supplement: Figure S2 — Cytokine response in splenocytes culture supernatant from various immunized groups at post booster on different time points. Archaeosome mediated Th1/Th2 polarization was ascertained by determining various cytokines at different time point post booster; (A) IFN-γ, (B) IL-12. To determine the antibody response IgG2a to IgG1 ratio was depicted using sandwich ELISA method (C). The data represent mean of three determinants± S.D. and are representative of two different experiments with similar observation. Statistically two groups were compared using t test analysis with p<0.05, p<0.01, p<0.001 level of significance. PB stands for post booster. For IFN-γ and IL-12 archaeosome entrapped Rv3619c Vs BCG p<0.01(eight week post booster), p<0.05 (sixteen week post booster); IgG2a∶IgG1 ratio p<0.05 (four week post booster, eight week post booster) and p = not significant (12 week and 16 week post booster). (TIF) [file pone.0022889.s002.tif]
